# XRF analysis searching for fingerprint elemental profile in south-eastern Sicily tomatoes

**DOI:** 10.1038/s41598-023-40124-6

**Published:** 2023-08-23

**Authors:** Salvina Panebianco, Maria Grazia Pellegriti, Claudio Finocchiaro, Agatino Musumarra, Germana Barone, Maria Cristina Caggiani, Gabriella Cirvilleri, Gabriele Lanzafame, Alfredo Pulvirenti, Agata Scordino, Paolo Mazzoleni

**Affiliations:** 1 Dipartimento di Fisica e Astronomia, Università di Catania, Catania, Italy; 2 Dipartimento di Agricoltura, Alimentazione e Ambiente, Università di Catania, Catania, Italy; 3 Istituto Nazionale di Fisica Nucleare, Sezione di Catania, Catania, Italy; 4 Dipartimento di Scienze Biologiche, Geologiche e Ambientali, Università di Catania, Catania, Italy; 5 Dipartimento di Medicina Clinica e Sperimentale, Unità Bioinformatica, Università di Catania, Catania, Italy; 6 Istituto Nazionale di Fisica Nucleare, Laboratori Nazionali del Sud, Catania, Italy

**Keywords:** Environmental sciences, Characterization and analytical techniques

## Abstract

The implementation of analytical techniques able to certify food quality and origin in a fast and non-destructive way is becoming a widespread need in the agri-food sector. Among the physical non-destructive techniques, X-ray fluorescence (XRF) spectrometry is often used to analyze the elemental composition of biological samples. In this study, X-ray fluorescence (XRF) elemental profiles were measured on tomato samples belonging to different geographical areas in Sicily (Italy). The purpose of this investigation was aiming to establish a protocol for in-situ measurement and analysis able to provide quality assessment and traceability of PGI agri-food products, specifically sustaining health safety and self qualifying bio-chemical signature. In detail, sampling was performed in one of the most tomato productive area of south-eastern Sicily (Pachino district), characterised by a relative higher amount of Organic Carbon and Cation Exchange Capacity, and compared with samples from other growing areas of Sicily, falling in Ragusa province and Mt. Etna region. Experimental data were analyzed in the framework of multivariate analysis by using principal component analysis and further validated by discriminant analysis. The results show the presence of specific elemental signatures associated to several characterizing elements. This methodology establishes the possibility to disentangle a clear fingerprint pattern associated to the geographical origin of an agri-food product.

## Introduction

Food nutritional and organoleptic characteristics, as well as food origin and hygienic-sanitary conditions, are fundamental requirements for quality assessment and safety, with a direct impact in healthy eating and social wellness.

Identity and origin of a product from a peculiar and valuable geographical area are recognized by the European Community with the labels “Protected Geographical Indication” (PGI) and “Protected Designation of Origin” (PDO). These labels aim to safeguard the names of specific products to promote their unique characteristics connected with their geographical origin.

It is already known in literature that the presence in vegetables of various bioactive compounds such as flavonoids, tannins and other polyphenolic constituents^1,2^ as well as of aromatic compounds, which are mainly responsible for the organoleptic properties of food, often identifies the product genuineness and healthiness. These are strictly related to the specific features of the territory of origin. Thus, checking food origin by determining the characterizing elements becomes extremely important for traceability and in fighting food fraud during the whole production, processing and marketing chain. In this context, food authentication is especially necessary for branded products, like PDO and PGI labelled ones, which are more susceptible to fraud than other products, due to their higher economic value.

The characterizing and trace elements in biological and environmental samples are usually identified by using traditional methods, such as atomic spectrometry techniques, including ICP-OES (Inductively Coupled Plasma Optical Emission Spectrometry) and ICP-MS (Inductively Coupled Plasma Mass Spectrometry)^3^. Despite the widespread use of the classical analytical instruments, non-destructive techniques have recently been introduced, with the double advantage to speed up the analysis and to avoid damages to the analyzed biological matrix. Moreover they require a minimum sample amount and can be used for online measurements at various stages of the industrial processing. Among these, the X-Ray Fluorescence (XRF) spectrometry is often used. It allows to identify and quantify the elements of a homogeneous or not homogeneous sample (solid or liquid) by measuring the fluorescent X-ray emitted from atomic shell de-excitation, following a primary X-ray irradiation (see^4^ and reference therein). XRF spectrometry has the advantage of being a relatively cheap technique, easy to implement (also on the field) and very fast, since it allows to determine presence and concentrations of lots of chemical elements in few minutes, with high accuracy and precision. However, this technique presents a great difficulty in determining chemical elements having an atomic weight lower than that of Na (Z $\leqslant$ 11). As a matter of fact, the incident X-ray beam is mainly scattered and poorly absorbed by organic samples particularly rich in light elements, such as C, H, O and N. In this case, a more extensive knowledge of chemical composition of a sample could be achieved by working in other spectral regions and using scattering-based spectrometric techniques, such as Rayleigh, Compton and Raman ones.^5,6^.

The present work aims to determine a reliable and fast procedure based on a portable XRF device to reveal “fingerprint” patterns, identifying the provenance of fresh and dried vegetables, in order to establish a reproducible analysis protocol, supporting alimentary certification and traceability. Recently XRF spectrometry, in combination with multivariate analysis of the elemental fingerprint patterns, has been successfully used to verify the geographical origin of different products, including paprika powder, honey, soybean seeds and extra virgin olive oils^7–13^, to discriminate organic foodstuffs from conventional ones, verifying the agronomic production system^14^, and to authenticate similar food commodities (sugars, oils) originating from different vegetable crops^13,15^. In this study, the XRF analysis preliminary validated in^4^ was applied to detect the presence of characterizing and trace elements in cherry tomato fruits (*Lycopersicon esculentum* L.) cultivated in different greenhouses falling into the Pachino district, an area located in the south-east of Sicily (Italy), in the provinces of Syracuse and Ragusa, that includes the entire municipality of Pachino and Portopalo di Capo Passero and part of the territories of Noto and Ispica.

In a preliminary step of this study we experimentally verified the reliability and robustness of the experimental method and analysis. The validation of the methodology was performed by tomato samples collected in the Pachino district (PGI sites). The elemental profiles and yields obtained by different XRF apparatus set-up (use of different filters) and sample preparation methodologies (presence/absence of tomato skin in the samples) were compared with each other in the framework of multivariate analysis (Principal Component Analysis, PCA) by using raw XRF spectra and the element yields, improving and simplifying the analysis. The innovative approach of a direct spectral analysis by PCA was validated, together with its robustness, with respect to the different experimental/sample conditions. This opens the possibility to implement an unsupervised analysis procedure to be used by non specialized operators directly on the field or along the chain of distribution, establishing a breakthrough in the agri-food certification methodology. Consequently, as a further step, XRF measurements were extended to tomato samples coming from greenhouses located in non-PGI sites of south-eastern Sicily and Etna region, in Ragusa and Catania provinces, in order to identify the elemental markers (within the categories of macro- and micro-elements) able of discriminating PGI tomato fruits within the full data set. Fingerprint markers were disentangled in association with the production area. Finally, the differences in tomato elemental composition were further investigated through the Discriminant Analysis (DA) and correlated to the soil physico-chemical properties.

**Figure 1:**
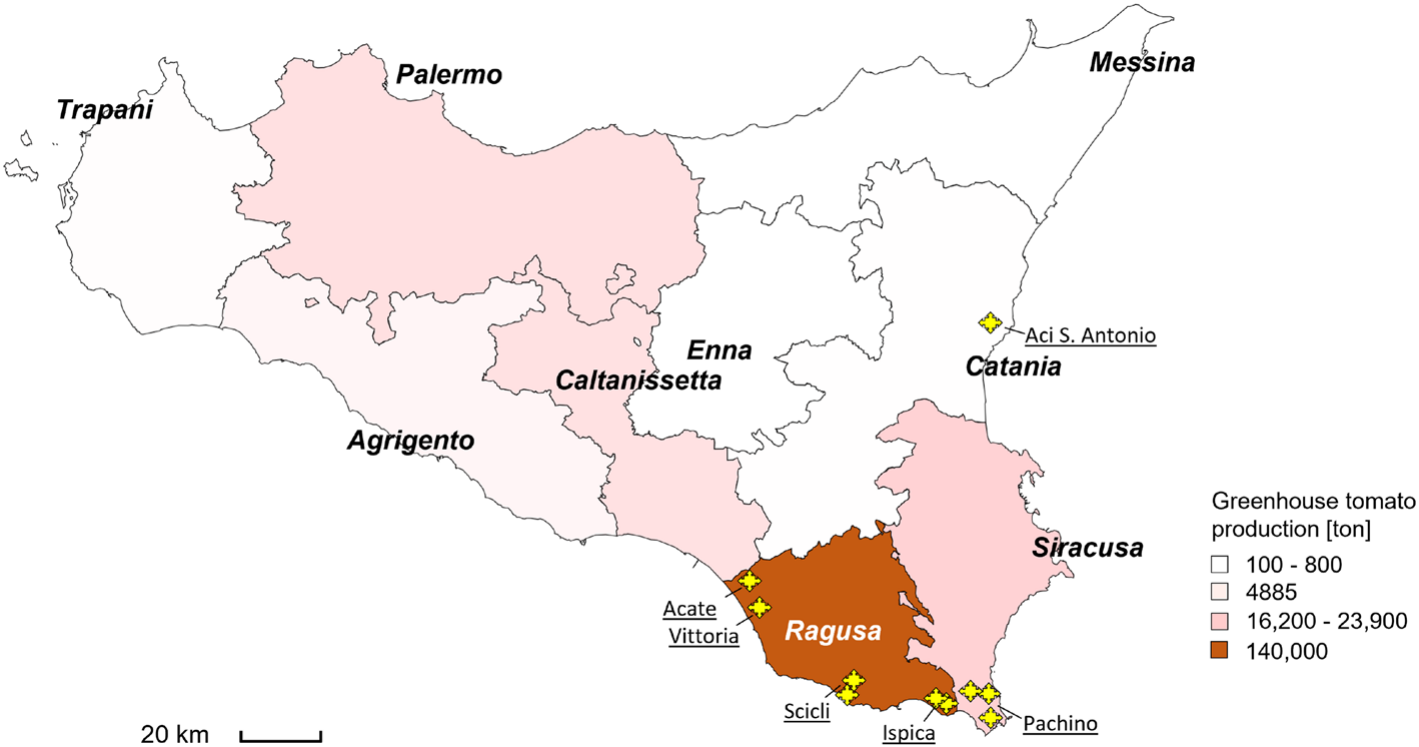
Tomato greenhouse production distribution within Sicily in 2022^16^ (production distribution in 2021 is reported in^17^). Province of Ragusa has the highest tomato production rate. Yellow stars indicate the sampled sites of the present study. The figure has been created by using Microsoft Excel Bing Maps, Bing $\copyright$, GeoNames, Tom Tom (https://www.geonames.org).

## Area of origin and sample characteristics

In Sicily, fresh market tomato is a major greenhouse vegetable crop, covering over almost 3038 hectares with a total production of 203,223 tons^16^ (Fig. 1 and^17^). Approximately 80% of the Sicilian greenhouse tomato production is located in the south-east of Sicily, in Ragusa and Syracuse provinces, known worldwide for the production of PGI Pachino tomato. Due to its strong connection with the peculiar features (soil and irrigation) of its territory, it was the first Italian tomato to obtain the European PGI certification of quality.

The peculiar nutritional and organoleptic features of PGI tomato of Pachino result from the combination of various environmental factors (pedological and microclimatic conditions, cultivation techniques, saltwater irrigation), which differ with respect other Sicilian geographical areas devoted to tomato crops. The area of origin of the present sampling campaign is reported into Fig. 1. Tomato fruits with PGI certification were collected from five farms located in Pachino and Ispica municipalities, in Syracuse and Ragusa provinces. In these farms, tomato plants were grown on unheated glasshouses assembled by using plastic films and they were watered by drip-irrigation, by using groundwater with a salinity level ranging from 1500 to 10 000 $\upmu$S/cm, according with the standard agronomical practices^18^. As concerning tomato fruits without PGI certification, they were harvested from five farms located in Acate, Scicli, Vittoria and Aci St. Antonio municipalities, in Ragusa and Catania provinces. In these farms, tomato plants were grown on unheated greenhouses covered with polyethylene sheets, by using an irrigation water with a salinity level lower than the Pachino district.

Portions of dried pulp with and without skin were prepared for XRF analysis according to the procedure used in^4^. A total of 24 samples belonging to three typologies (cherry, plum and miniplum) and to eight cultivars of tomato (Creativo, Ciringuito, Lavico, Mozia, Paskaleto, Pixel, Prodigio and Rokito) were analyzed. Samples were harvested at full ripeness stage (fully red skin), as usually occurs for marketing. Table 1 summarizes geographical origin, tomato typology and cultivar of the samples.

Microbiological analyses were also performed on tomato samples harvested in the Pachino district during the same campaign^19^.

**Table 1:** Experimental campaign: sample origin (origin), farm, greenhouse (GH), tomato typology (Typ.), cultivar (Cv.), site typology (site), sample identifier (ID), measurements M1 (filter Ti/Al/Cu and “pulp” sample), M2 (Ti/Al filter and “pulp” sample) and M3 (Ti/Al filter and “pulp + skin” sample).

Origin	Farm	GH	Typ.	Cv.	Site	ID	Measurement
Pachino (SR)	1	1	Cherry	Paskaleto	PGI	A1, A2, A3, A4	M1, M2 and M3
Pachino (SR)		2	Plum	Pixel	PGI	P1	M1, M2 and M3
Pachino (SR)		3	Plum	Pixel	PGI	P2	M1, M2 and M3
Pachino (SR)	2	4	Cherry	Mozia	PGI	B1	M1, M2 and M3
Pachino (SR)		5	Cherry	Mozia	PGI	B2	M1, M2 and M3
Pachino (SR)	3	6	Cherry	Ciringuito	PGI	C1	M1, M2 and M3
Pachino (SR)		7	Miniplum	Rokito	PGI	M1	M1, M2 and M3
Ispica (RG)	4	8	Cherry	Creativo	PGI	D1	M1, M2 and M3
Ispica (RG)		9	Cherry	Creativo	PGI	D2	M1, M2 and M3
Ispica (RG)	5	10	Cherry	Lavico	PGI	L1	M1, M2 and M3
Ispica (RG)		11	Cherry	Lavico	PGI	L2	M1, M2 and M3
Acate (RG)	6	12	Cherry	Prodigio	non-PGI	AC1	M3
Acate (RG)		13	Cherry	Prodigio	non-PGI	AC2	M3
Scicli (RG)	7	14	Cherry	Mozia	non-PGI	SC1	M3
Scicli (RG)		15	Cherry	Mozia	non-PGI	SC2	M3
Scicli (RG)	8	16	Cherry	Mozia	non-PGI	SC3	M3
Scicli (RG)		17	Cherry	Mozia	non-PGI	SC4	M3
Vittoria (RG)	9	18	Cherry	Prodigio	non-PGI	V1	M3
Vittoria (RG)		19	Cherry	Prodigio	non-PGI	V2	M3
Aci St. Antonio (CT)	10	20	Miniplum	Rokito	non-PGI	ET1, ET2	M3

In farm n. 1, four cherry tomato samples (cv. Paskaleto) were collected in greenhouse 1, the largest one, made up of 120 rows of plants. The other greenhouses were smaller and housed plants arranged in short rows, ranging between 20 and 30.

## XRF measurements and analysis

XRF measurements were performed by using the Bruker portable spectrometer (Tracer IV-SD) as in^4^. The handheld spectrometer, equipped with an X-ray tube (Rh anode) having a X-ray beam collimated at 3 mm in diameter, was set to operate at an anodic voltage and current of 40 kV and 17 $\upmu$A, respectively. For all the tomato samples, measurement time was set to 120 s.

The reliability and robustness of the measurement were tested on a sample subset (PGI samples, Table 1) by coupling two different filters to the apparatus, Ti/Al/Cu and Ti/Al, and by preparing two dried sample typologies, “Pulp” and “Pulp + Skin”. Then, the measurements were extended to the full sample set (PGI and non-PGI samples, Table 1), implementing only the Ti/Al filter and “Pulp + Skin” sample typology.

XRF spectra were acquired by using the S1PXRF software and the fit was performed by using the PyMca software^20^. Before the fitting procedure, spectra were energy calibrated. The peaks associated to the K$\alpha$, K$\beta$ and L X-ray transitions, characterizing each element, were fitted by the Mca Hypermet fit function. Background subtraction was performed through the PyMca “strip” algorithm by using a width of 25 channels; reduced $\chi ^2$ values of the fit were ranging from 1.02 to 1.48.

In order to assess the best methodology in disentangling tomato samples according to their geographical origin, three different data-sets were obtained from each measurement on a given sample which were subsequently used in the statistical analysis: “original spectrum”, including background signal, “net spectrum”, without background component and “element yields”, net peak integrals associated with characterizing and in-trace elements.

### Reliability test

**Figure 2:**
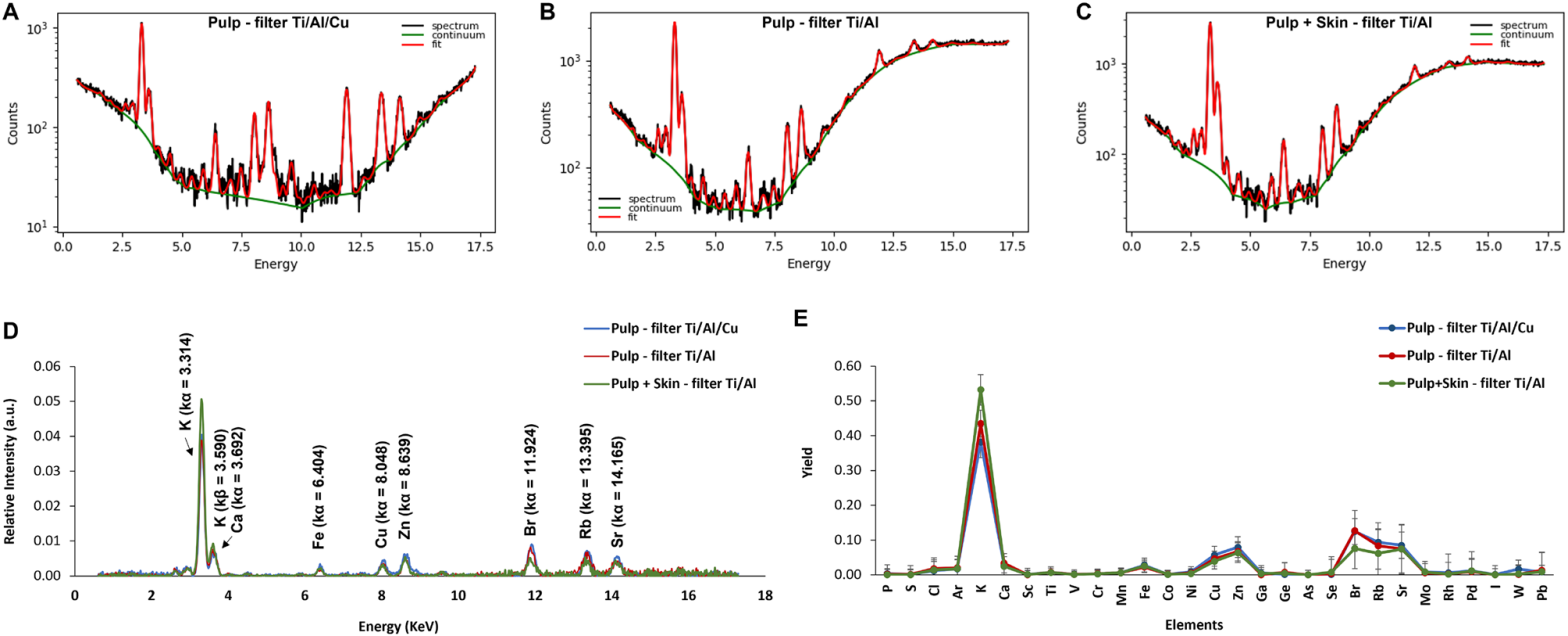
XRF spectra associated to the “pulp” and the “pulp + skin” of D1 sample. The spectra referred to the “D1—pulp” sample, obtained by configuring the Bruker spectrometer with Cu/Ti/Al (0.006” Cu, 0.001” Ti, 0.012” Al) and Ti/Al (0.001” Ti, 0.012” Al) filters, are plotted in (**A**) and (**B**), whereas XRF spectrum associated to “D1—pulp + skin” sample, measured with Ti/Al filter, is plotted in (**C**). In (**A**), (**B**) and (**C**), the raw spectra are reported in black, data global fit and background evaluation in red and green, respectively. In (**D**) the comparison of XRF spectra after background subtraction for the “pulp” of D1 sample obtained by using Ti/Al/Cu and Ti/Al filters (blue and red line, respectively) is shown; in the same plot, net XRF spectrum for the “pulp + skin” of D1 sample measured with Ti/Al filters is reported as a green line. Finally, plot (E) reports the extracted element yield distributions concerning the three experimental configurations M1, M2 and M3. Statistical error bars are included.

The performances of the XRF apparatus were studied by making use of the two different filter configurations in order to validate the robustness of the measurement with respect to the experimental conditions. For this purpose, the “Pulp” samples were irradiated by an X-ray beam hardened by Cu/Ti/Al (0.006” Cu, 0.001” Ti, 0.012” Al) (measurement M1, Table 1) or Ti/Al (0.001” Ti, 0.012” Al) filters (measurement M2, Table 1). Raw spectra belonging to the same sample (pulp of sample D1) obtained after irradiation by the two filters are shown in Fig. 2A,B. As expected, the spectra exhibit different backgrounds, in particular at X-ray energies larger than 7.5 keV. The main background component is originating from Compton and Rayleigh scattering. A further background component is due to the interaction between the characteristic X-rays of the sample and the detection instrument, including internally generated instrument noise. It is worthwhile to stress that, especially in the detection of trace elements, the background signal may have a significant effect on the detection limit and precision; thus, performing the same measurement with different background is crucial to assess that the spectroscopic information is preserved in spite of different experimental conditions.

For each sample, the net spectra (after background subtraction) have been normalized to the sum of the counts, while the obtained element yield distributions have been normalized to the total sum of the yields. The results are reported in Fig. 2D,E, respectively. Negligible differences, within the error bars, appear for the two filter configurations (see blue and red lines in Fig. 2D,E). This indicates that the choice of the filter used in the XRF apparatus does not significantly affect the obtained element distribution, thus validating the overall fit and background subtraction procedure.

A further analysis of the elements profile was performed with respect to the change of the sample texture by comparing the XRF measurements obtained from the “Pulp” samples (measurements M1 and M2) with those obtained from the “Pulp + Skin” ones (measurement M3, Table 1). In this way, it was possible to evaluate differences on the deduced element pattern related to the sample preparation methodology and to the skin presence. XRF measurements were performed on the “Pulp + Skin” samples by using the Ti/Al filter. The analysis procedures, curve fitting, background subtraction and elemental identification in the “Pulp + Skin” samples spectra were carried out by the same procedure previously described (Fig. 2C,D,E). Element profiles of the “Pulp + Skin” samples agree within the error bars with those obtained by using “Pulp” ones.

The elemental profiles of the three sets of measurements M1, M2 and M3, obtained by using the two filters and the two sample preparation methodologies used for the PGI samples (Table 1), were then analysed by the Principal Component Analysis (PCA) as implemented in the MATLAB software package^21^. The multivariate statistical analysis is usually associated to spectroscopic techniques, as it highlights correlations and/or differences among data-sets^22–24^. In order to disentangle differences introduced by using different analysis methodologies, for each set of measurements, PCA was performed on the three derived data-sets described above: raw XRF spectra, net spectra (background subtracted) and element yield distributions.

The first two PCA data matrices (size 14x840), obtained from raw and background subtracted spectra, were built by simply reporting in the rows the counts corresponding to the ADC channels (for a total number of 840 channels) and labelling the columns by the fourteen PGI tomato samples, originating from Pachino and Ispica growing areas. The third data matrix (size 14 $\times$ 29) was built by reporting in the rows the yields of the 29 obtained elements extracted after the fit and background subtraction procedure. The PCA was performed on the standardized matrices.

**Figure 3:**
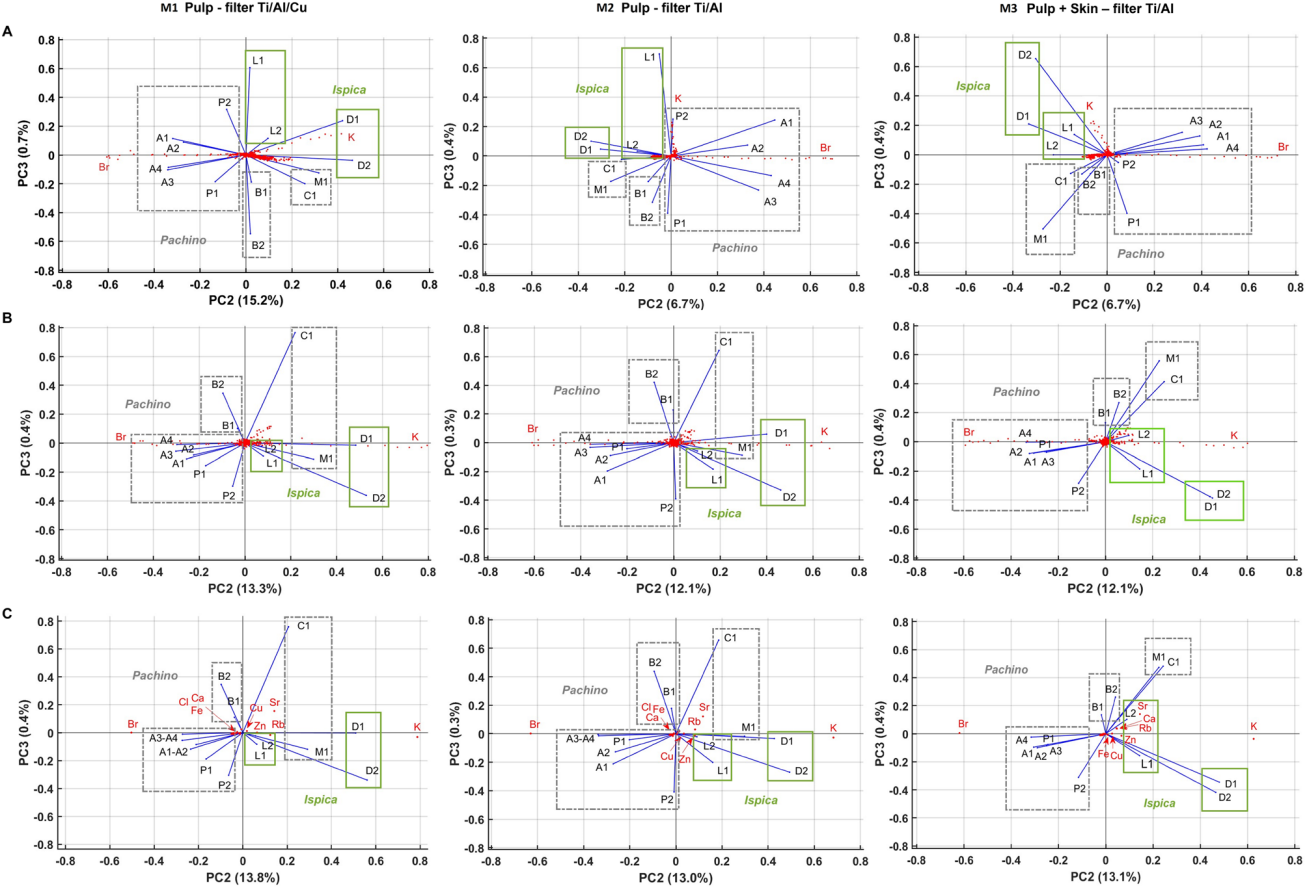
Comparisons of PC3 versus PC2 biplots obtained by analyzing “original spectra” with background (**A**), “net spectra” without background (**B**) and “element yields” (**C**) of “pulp” samples irradiated by an X-ray beam reduced by using Ti/Al/Cu filter (plots in the left side), Ti/Al filter (plots in the center) and of “pulp +skin” samples and Ti/Al filter (plots in the right side). Samples were obtained by PGI-tomatoes coming from the Pachino district (Pachino and Ispica municipalities). The variables, represented trough vectors, are the tomato samples collected from Pachino (samples A1, A2, A3, A4, P1, P2 from farm 1; B1, B2 from farm 2; C1, M1 from farm 3) and Ispica (D1, D2 from farm 4; L1, L2 from farm 5) (for details see Table 1).

As expected, for all the evaluated data-sets, the highest percentage of the total variance is associated to the first Principal Component (PC1) which ranges from 83.5% to 92.6% and, consequently, it hardly contains information for discriminating among the investigated tomato growing areas. On the other hand, large PC1 values confirm the overall data set consistency related to the average elemental composition of the tomato fruit; this overall consistency was further confirmed also in^4^ by using different analytical techniques and data-sets. Remarkably, PC2 and PC3 values make, instead, possible to disentangle tomato samples by their provenance, as the data set clustered into different groups related to the farms. The biplots of the second and third components (PC2 vs PC3) extracted by the three different data-sets were compared in Fig. 3A,B,C for the three sets of measurements M1 (left side), M2 (center) and M3 (right side). All the nine biplots shown in the figure, although obtained from three different data-sets, derived in turn from two different filter configurations and two sample preparation methodologies, clearly show five different clusters, which correspond to the five different farms surveyed within the Pachino district. Moreover, as it can be seen by the elemental analysis (Fig. 3), K is the most characterizing element in samples collected in the territory of Ispica (farms 4 and 5), while Sr and Rb mainly characterize the tomato collected in farm 3 located in Pachino. Finally Br is the dominant element in the tomatoes grown in farm 1 located in Pachino.

In this framework, it is important to remark that the results highlighted by the nine PCA analyses are fully consistent with each other (Fig. 3) and they do not vary significantly as a function of the filter used for the setting of the spectrometer, the texture of the sample or the spectrum analysis methodology. This confirms, from one side, the reliability of the XRF technique in detecting the chemical elements in a sample regardless of the instrumental conditions and the correctness of the fitting procedure and background subtraction carried out on the spectra. From the other side, it suggests the possibility of discriminating the samples on the basis of their “elemental profile” by a direct information retrieval based on the bare spectrum, bypassing the procedure of background subtraction and spectroscopic fit determining the element yields. This unique analysis procedure, which represents a step forward with respect to the work presented in^4^, has the further advantage of preserving all the information contained in the spectrum, often defined by the trace elements present in such small quantities that they cannot be disentangled from the background. The pragmatic consequence leads to consider just the raw spectra as the most meaningful and complete pattern in order to perform a quick and easy authentication. This kind of analysis protocol can be extended well beyond the biological case study.

Concerning the PCA made on “Pulp + Skin” samples, it confirmed the results previously highlighted by PCA carried out on the corresponding “Pulp” samples, although some differences were unveiled concerning the classification of the samples into groups. Specifically, samples of “Pulp + Skin” (Fig. 3, biplots on the right side) appeared to achieve a better discrimination than the samples of “Pulp” without skin (biplots on the center). Indeed, as shown in the right side of the Fig. 3, the clustering obtained by using the “Pulp + Skin” samples persisted even after the subtraction of the background and in the analysis of the element yields (see for instance the different position of the vectors M1 and C1 in the biplots representing the “Pulp” and the “Pulp + Skin” samples). Due to the presence of the skin, it is also possible to reveal a clear discrimination of the farms located in Pachino with respect to those located in Ispica, since they are populating different quadrants in the PC3 versus PC2 biplots. This result is related to the skin trace elements content; in fact, Ca and Sr are present in smaller quantities in the pulp. To this regard, as expected, the skin appears to be a valuable constituent when authenticating a food product. The analysis of fruit not subjected to peeling procedure provides important information that could be used for subsequent studies, laying the foundations for more extensive XRF applications, to be carried out directly in the field or on fresh market fruit.

### Full sample-set measurements and analysis

In the final step of this study, XRF measurements were extended to the non-PGI tomato samples originating from Ragusa and Catania provinces. Since, as previously demonstrated, the “Pulp + Skin” samples provide a better discrimination, requiring also shorter preparation times, the subsequent analyses regarding the non-PGI tomato samples were performed exclusively on the “Pulp + Skin” samples. Experimental data including the data-sets of tomato samples collected in the Pachino district and ten non-PGI tomato samples coming from five farms, located in Acate, Scicli, Vittoria and Aci St. Antonio (see Table 1), for a total of 24 samples, were analysed by PCA. The PCA data matrix (size 24 $\times$ 840) was built by reporting—in the rows—the ADC counts and—in the columns—the twenty-four tomato samples coming from Syracuse, Ragusa and Catania provinces. Statistical analysis was performed on the bare spectra data-set, containing also information of trace elements that cannot be disentangled from the background. As expected, being the samples of the same nature, with similar degree of ripeness and obtained by the same drying procedure, most of the information from XRF data is included in the first two PC. In detail, PC1 alone explained 93.2% of the total variance and PC2 explained 5.7%; consequently, to discriminate the different investigated tomato growing areas, PC3 and PC4 values have to be used (Supplementary Fig. S1, left side). Moreover, it was observed that the samples from the first greenhouse of farm 1 in Pachino (A1–A4) were lying between the non-PGI samples collected in the province of Ragusa, far away from the PGI-samples group, preventing the formation of two distinct groups (Supplementary Fig. S1,B,C).

The peculiar position assumed by A1–A4 samples in the different biplots must be attributed to the dominant presence of Br element (such that can be directly inferred by inspecting the XRF spectra), whose concentrations differ significantly with respect to the value deduced for the other samples. In order to proceed to a data rejection for A1–A4 samples, we infer that the high yields of Br found in the samples from the greenhouse n. 1 could be related to anthropogenic causes (specific soil treatment, use of particular pesticides before harvesting, etc.). This conclusion can be deduced also by our previous study concerning the epiphytic and endophytic microbial populations isolated from the samples A1–A4^19^. These samples, although coming from farm 1 in the Pachino area, separated from the other samples creating a third cluster. Thus, according to their large differences with respect to the other samples, they can be considered as outliers.

**Figure 4:**
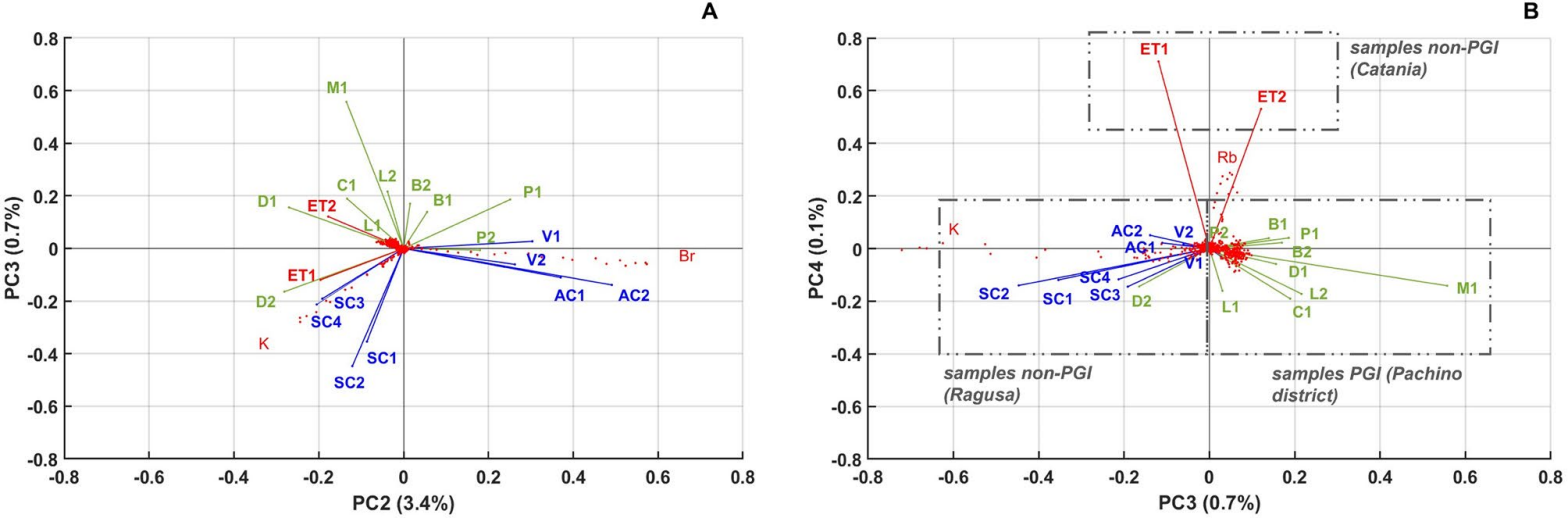
PCA carried out on “pulp + skin” samples obtained from tomatoes with PGI and non-PGI certification (in **A**, biplot PC3 vs PC2; in **B**, biplot PC4 vs. PC3). The biplots were obtained by the raw spectra data-sets, made excluding A1–A4 samples (outliers). The variables (vectors symbolizing tomato samples coming from PGI- and non-PGI sites) were distinguished by using different colours (in green, PGI samples from Pachino district; in blue, non-PGI samples from Ragusa; in red, non-PGI samples from Aci St. Antonio).

For this reason, a new statistical analysis was performed excluding the samples collected from greenhouse 1, thus finding a full coherence in the context of the PCA. The results are shown in Fig. 4. Such results are also reported in Supplementary Fig. S1 (right side) to directly compare the clustering in presence and in absence of the outliers.

In all new biplots, most of the tomato samples coming from the same farm were located close to each other. Moreover, excluding A1–A4 tomato samples from greenhouse 1, samples originating from the Pachino district (PGI samples) and from Ragusa and Catania provinces (non-PGI samples) were well separated from each other by the PCs of higher order, as you can see in detail in the Fig. 4A,B. In particular, “Pulp + Skin” samples clustered into three distinct groups, linearly separated, based on their PC3 and PC4 values: the PGI-tomato samples coming from Pachino and Ispica are characterized by positive PC3 scores, the non-PGI samples from Ragusa province are characterized by negative PC3 scores, whereas the tomatoes produced around the Mt. Etna had again values with positive scores on PC4 (Fig. 4). As shown by PC3-PC4 biplot, exclusion of outliers leads to a full and coherent discrimination of the different geographical areas of origin. This result confirms the previous analysis reported in the feasibility study^4^ and suggests that the XRF technique is able to discriminate tomato coming from different geographical origins with reasonable accuracy. Since the measured XRF patterns can be associated to the specific features of the tomato growing areas (soil composition, agronomical practices and microclimatic conditions establishing inside the greenhouses), XRF analysis can be successfully used to assess the geographical origin and the quality of foodstuffs and, consequently, to detect counterfeit products.

These results were confirmed by a further analysis of the element yields obtained by the overall fit of the spectra (see Supplementary material, Fig. S2), as previously done in Fig. 3. However, in this case, the clustering was less evident, due to the effect of the raw data filtering. Only the tomato samples from Aci St. Antonio (Catania) kept their position unchanged, remaining well apart from all the others. So, it is possible to infer, as expected, that the background subtraction resulted in a loss of information. All in all, elemental differences of tomato samples from Ragusa, Ispica and Pachino are sometime revealed by the presence of trace elements in such a small quantities that, having an unfavorable signal/background ratio, cannot be disentangled by using the fitting procedure and, therefore, are washed out by the background subtraction.

## Element analysis and relationship with soil characteristics

As already stated in the introduction, the peculiar nutritional and organoleptic features of a foodstuff are the result of various external environmental factors, including pedological and micro-climatic conditions and cultivation techniques.

Considering the pedological conditions, it is worthwhile to stress that soil properties influence the biochemical composition and the bio-availability of soil trace elements, affecting their absorption and accumulation in plant tissues. Moreover, concentrations of trace elements in the soil are influenced by the layer in which they develop and by the lithology of pedogenic rock^25^. Therefore, different plants grown on different geological types of rock and on different soils may exhibit different chemical composition. In this context, accumulation rates of elements have been found correlated, in fruit and wine samples, with the soil composition of different geographical areas^25–27^.

Given that premise, assuming the existence of strong interrelationships within the plant-soil system, we started investigating on the origin of the different chemical composition of the analyzed tomatoes, evaluating the characteristics of the Sicilian soil, in particular for Catania, Ragusa and Syracuse provinces. To this end, we used the ‘Land Use and Coverage Area frame statistical Survey Soil’, LUCAS, database^28^, which reports the physical and chemical properties of soil samples collected in the European Union^29^. In detail, data related to the elemental composition of tomato samples can be correlated with some physical and chemical properties of soil^29^, namely Soil Texture, Calcium Carbonate (CaCO$_3$) content, Organic Carbon (CO) content, Cation Exchange Capacity (CEC), Soil pH in water, Nitrogen (N) content and Extractable Potassium (K) content as reported in the LUCAS Soil database, by considering the same surveyed geographical areas (sampling sites).

Before proceeding to fruit-soil comparison, the local changes in tomato elemental composition, previously highlighted by PCA, were further evaluated and validated through supervised multi-parametric analysis tools (Discriminant Analysis). For this purpose, the element yields previously extracted from XRF spectra associated to the tomato “Pulp + Skin” samples were analysed, excluding the samples collected from the greenhouse n. 1 (considered outliers as described in the previous paragraph; see Table 1). The XLSTAT package^30^ was used in order to perform the Discriminant Analysis (DA). The input data matrix was reporting—in the columns (variables)—the element yields associated to nine elements, previously normalized to the Ar yield, and labelling the rows (observations) by the twenty tomato samples originating from the different surveyed farms (see Table 1). The nine elements reported in the matrix were selected in order to have the yield ratio to Argon greater than one, consequently the dimension of the input data matrix was 9x20. Since it is supervised, DA requires a class membership assignment for each sample. So, in order to proceed with the analysis, six different classes corresponding to the six different surveyed municipalities—Pachino, Ispica, Acate, Scicli, Vittoria and Aci St. Antonio, see Table 1—were defined; subsequently, each tomato sample was labelled according to its class, respecting the geographical area of provenance.

**Figure 5:**
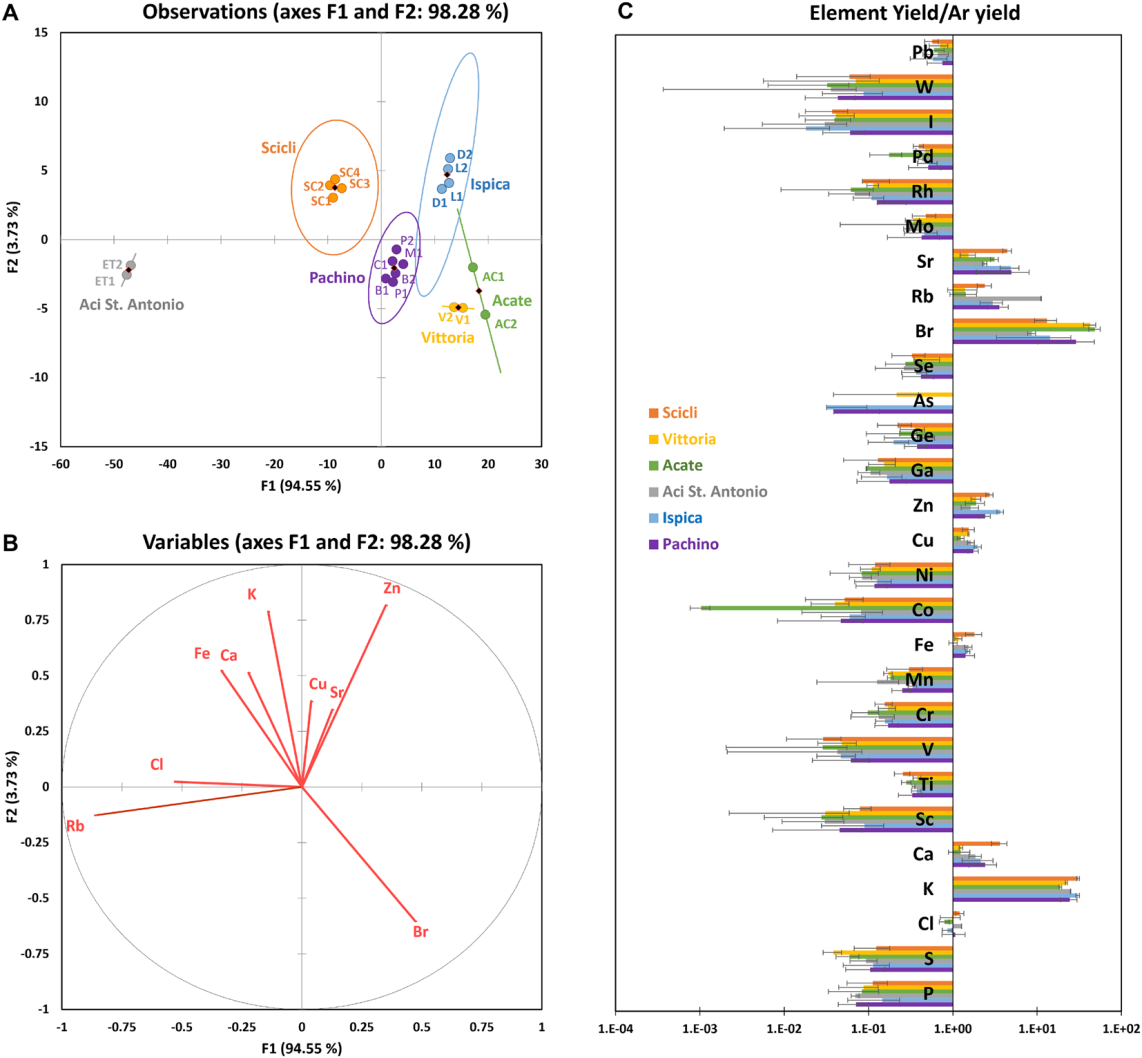
On the left, DA plots obtained by using nine variables including the elements with larger yield (element to Argon ratio larger than one): observations are plot in (**A**) and variables are plot in (**B**). In (**C**), averaged element yields detected in the “pulp + skin” samples obtained from tomatoes with PGI and non-PGI certification harvested in Pachino (P1, P2, B1, B2, C1, M1), Ispica (D1, D2, L1 and L2), Scicli (SC1, SC2, SC3 and SC4), Vittoria (V1, V2), Acate (AC1, AC2) and Aci St. Antonio (ET1, ET2). Yields have been normalized to Argon yield for each region before being averaged. In correspondence of each element, bars show the standard deviations.

The discrimination of tomato samples (observations) achieved on the basis of the first two discriminant functions and the contribution of each element (variables) in discriminating the analyzed samples are reported in Fig. 5A,B, respectively. In addition to DA plots, Fig. 5C shows the averaged “element yields to Ar yield ratio” obtained for each geographical area (the class). The correlation matrix between the used variables, the classification functions and the group comparison after the DA statistical analysis, which highlight that all observations were correctly classified, are shown in Supplementary Table S1, S2 and S3. As expected, DA correctly discriminates the sample clusters in relation to their geographical origin (Fig. 5). The two discrimination functions described 94.6$\%$ (F1) and 3.7$\%$ (F2) of the total variance, respectively. The cluster discrimination is fully consistent with the PCA biplots shown in Fig. 4. As shown by the DA plot reporting the observations, six different clusters are fully disentangled by using the first two discrimination functions, which correspond to the six different growing areas surveyed (Fig. 5A). Tomatoes from Aci St. Antonio showed the greatest differences in terms of elemental composition compared to the tomato average composition, as they are placed far away from the plot center and from the other five groups. On the opposite, the two classes concerning tomatoes harvested in Pachino and Ispica, as well as those representing the tomatoes from Acate e Vittoria were close to each other, indicating that their elemental profiles are similar. The squared distance values reported in Supplementary Table 3 show, in detail, how the observations distribute around each group mean value. This result agrees and further confirms PCA results, which clearly discriminated between PGI and non-PGI samples (see PC3 vs PC4 in Fig.  4B and 5A). The variables involved to a greater extent in discriminating tomato samples according to the geographical origin were mainly Rb, Br and Zn, whereas Cu, Sr and Cl showed the least ability in discriminating (Fig. 5B). In conclusion, as previously found in the feasibility study^4^, the most significant XRF yields were obtained for K, Ca, Fe, Cu, Zn, Br, Rb and Sr elements. The tomato samples coming from Scicli and Pachino district (Ispica and Pachino) differed from the other samples for the greater content of the elements Ca, Zn and Sr. Finally, a strongly characterizing content of Rb was found in the samples collected at the foot of Mt. Etna (Fig. 5B,C).

**Figure 6:**
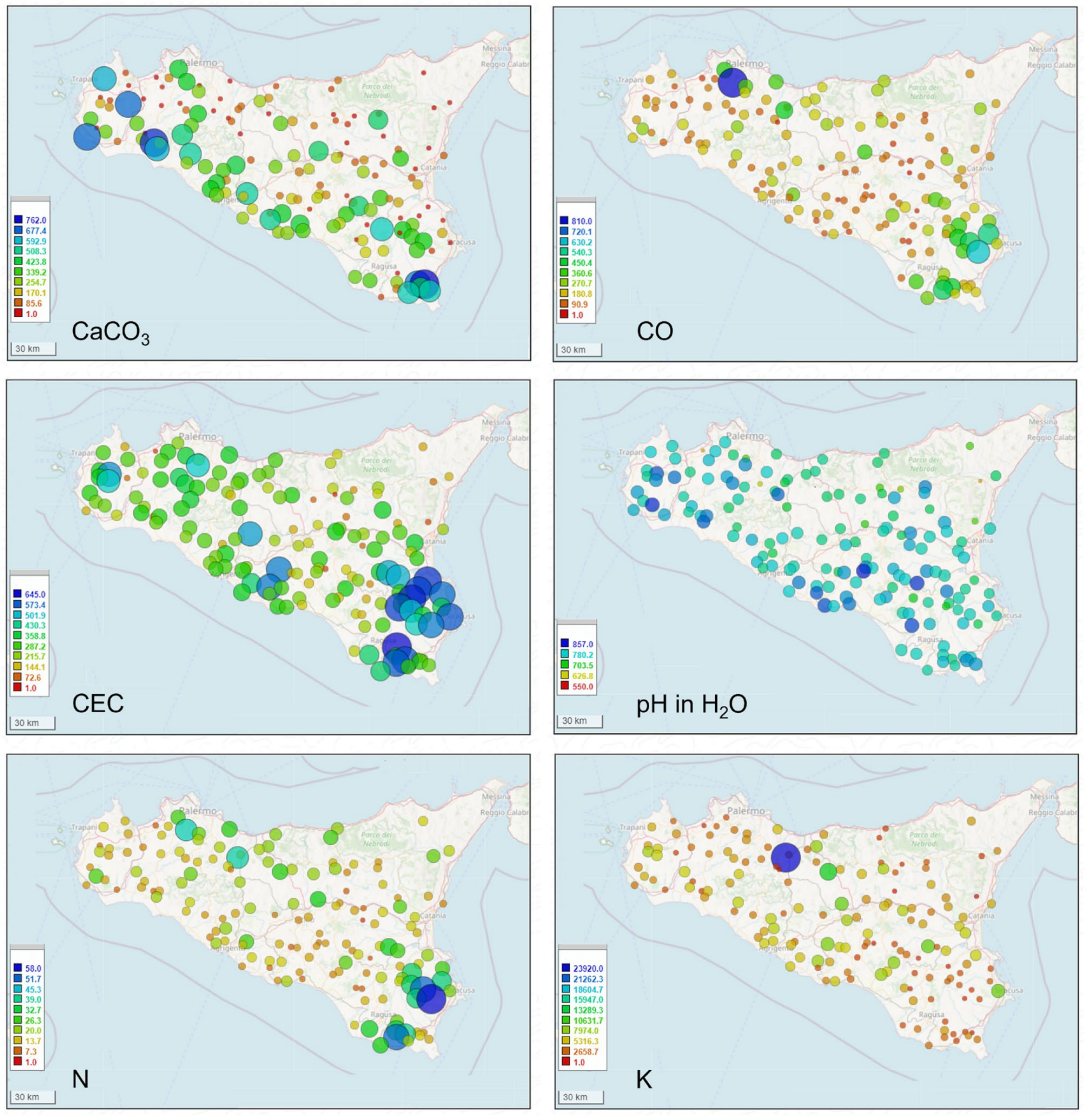
Bubble maps extracted from LUCAS database for Sicily concerning the values of CaCO$_3$ (g/kg), CO (g/kg), CEC (cmol(+)/kg), pH in H$_2$O, N (g/kg) and K (g/kg) in soils. Maps have been created by using the software GPS Visualizer (https://www.gpsvisualizer.com)^35^. Map data were obtained from OpenStreetMap (https://www.openstreetmap.org).

**Figure 7:**
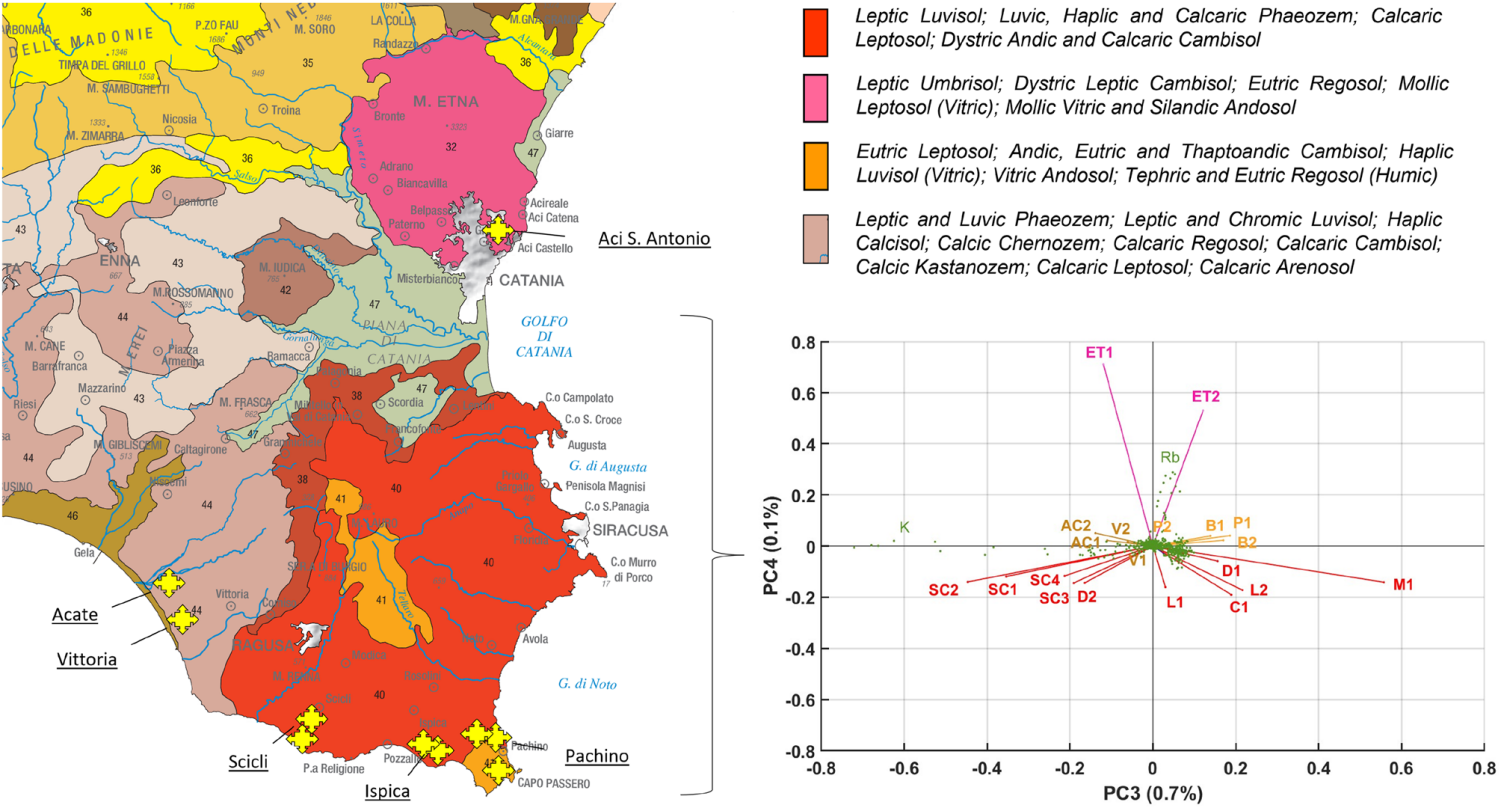
On the left, sampling sites (yellow stars) are overlapped on the pedological map from^31^; on the right, PCA results obtained from the full sample-set measurements (already shown in Fig. 4). In the biplot, the samples are plotted with the same color code used to indicate the pedological zones of provenance: fuchsia for ET1, ET2 samples (zone n. 32 in the map); red for SC1, SC2, SC3, SC4, D1, D2, L1, L2, M1, C1 samples (zone n. 40); orange for B1, B2, P1, P2 samples (zone n. 41); beige for AC1, AC2, V1, V2 samples (zone n. 44). Map reproduces, with appropriate modifications, the Soil Map of Italy reported in^31^.

As a final step of our study, in addition to the statistical analysis carried out on the element yields, we proceeded to qualitatively analyze some soil physico-chemical parameters characterizing the sampling sites under study, in order to evaluate possible correlations between the tomato elemental composition and the territory. The Sicilian soil properties were extracted by the LUCAS Soil database and are plotted in Fig. 6. The surveyed farms were also localized on a pedological map of Sicily^31^ (Fig. 7). Unveiling the complex biochemical motivations leading to a direct or indirect correlation between the fruit elemental profile and the soil characteristic lies well beyond the purpose of the present work. Here we are limiting the analysis to detect ex-post correlations (phenomenological) between the elemental profile and the soil composition for the six investigated sites on the basis of the parameters reported by the LUCAS database.

The growing areas falling in the Pachino district are characterized by clay-loam soils, with a prevalence of silt, high salinity conditions and favorable climatic conditions that permit extra seasonal productions of different vegetal crops. Moreover, the Pachino and Ispica farmlands are particularly rich in calcium carbonates (CaCO$_3$) and contain a moderate content of organic carbon (CO) (Fig. 6). The presence of free CaCO$_3$ generally reduces the solubility of elements in the soil, increasing the pH; moreover, bicarbonate ions form element carbonates sparingly soluble^32^. On the contrary, CO content improves the nutrient availability, the activity of soil microorganisms and the soil fertility^33^. Naturally-occurring organic carbon forms are linked to the soil organic matter, represented by undecomposed or partially decomposed plant and animal residues and by substances synthesized by soil organisms as a result of decomposition processes. Soil organic matter is made by different fractions, including simple molecules (amino acids, monomeric sugars), polymeric molecules (cellulose, protein, nucleic acids, lignin, etc.) and highly decomposed compounds such as humus^34^.

The farmlands under study falling in the Ragusa and Catania provinces, intended for the cultivation of tomato fruits without PGI certification, have different characteristics than those in the Pachino district: the farmlands in Scicli are mainly loamy, rich in N and CO content and poor in calcium carbonates, the ones in Vittoria and Acate are sandy-loamy and poor in N and CO content, while the soil around Mt. Etna is volcanic, with a prevalence of sand^28,29^. Some of these soil characteristics are reported in the Fig. 6.

The soils formed in volcanic areas have properties that clearly distinguish them from other soils of different origins: in particular, they have a variable electric charge and a high anion exchange capacity^36^. In addition, data processing from the LUCAS database highlighted in the Etna region a significantly lower content of calcium carbonates and organic matter than that found in other areas of Sicily (Fig. 6). The content of soil organic matter (expressed as CO concentration in g/kg of soil) and the humus fractions, such as humic and fulvic acids, as well as imogolite-type materials and crystalline Fe-oxyhydroxides, decrease with increasing altitude^37^, affecting the vegetation systems and the soil fertility.

As shown in Fig. 6, the growing areas monitored in this study differed from each other also for the cation exchange capacity (CEC), whose values were particularly high in the soils falling in the Pachino district and in the territory of Scicli. The cation exchange capacity (CEC) is a critical and dynamic component of soil chemical systems. It is considered an indicator of soil fertility, as it represents the total amount of exchangeable cations that the soil particles can retain on their surface in plant-available form (^38^ and references therein).The positively charged ions (cations), such as Na, K, Mg, Ca, Mn, Cu, Zn and Fe, are typically adsorbed and held on the negatively charges present on the surfaces of clay minerals and organic matter of soils by electrostatic forces and this allows exchanges with other positively charged particles in the surrounding soil water. The CEC values are influenced by pH and soil texture, as well as by the type of clay and the organic matter content; in detail, they are typically higher in soils with a high organic matter and clay content and under alkaline pH conditions^38,39^.

On the basis of these premises, it is reasonable to assume that the higher content of Ca, Zn and Sr elements found in tomato samples coming from farms sited in Pachino, Ispica and Scicli is due to the higher CEC and CO values characterizing the soil samples from these geographical areas, that increase the availability of elements in the soil. In this regard, it is known that a trace element will be accumulated in the plant according to a sigmoid-type function as its concentration in the soil is increased^40^. As for the tomato samples harvested in Mt. Etna, the content of some elements found in them, such as Rb, could be instead related to volcanic soil. In fact, as reported in literature, large amounts of Mn, Co, Ni, Pb, Rb, Zn, As, Cd, Ti and Se were reported in volcanic soils and in the groundwater system around the Mt. Etna; some of these elements, in particular the most volatile elements, derive from emission of gases and magmatic degassing, while others are constituents of hot magma and magmatic rocks^41–43^. The correlations between the elemental composition of tomatoes and their geographical origin were also highlighted by overlapping the sampling sites on the pedological map of Sicily reported by^31^ (Fig. 7).

As shown, it was possible to attribute the provenance of the tomato samples to four different pedological zones (Fig. 7, left side) which correspond to the four clusters revealed by PCA on the XRF spectra (Fig. 7, right side). To better visualize the correlation between the clustering obtained by PCA and the surveyed tomato growing areas, the vectors corresponding to samples were marked by using the same color legend present in the map.

Once again the overall chemical composition of fruit reflects the geochemical composition of the soil, as reported in literature^25–27^.

### Plant collection

This experimental research does not involve plant species at risk of extinction. Tomato samples for this study were kindly provided by farms’ owners, without any payment and formal request for the collection of plant material.

### Ethics statement

The collection and use of any plant materials in this study are carried out in accordance with EU and Italian guidelines and regulations.

## Conclusions

The XRF measurements and analysis carried out on tomatoes grown in different PGI and non-PGI sites confirmed the results achieved through the previous feasibility study, demonstrating that XRF analytical technique is a reliable tool to authenticate products based on their geographical origin. The measured XRF spectroscopic pattern can be associated to features of the tomato growing areas connected to soil composition, to company management and to microclimatic conditions in the greenhouses. The PCA, corroborated by DA, evidenced the presence of characterizing elements such as Cl, K, Ca, Fe, Br, Cu, Zn, Rb and Sr in all the analyzed samples, albeit at different concentrations. In particular, Rb characterizes the samples collected in the volcanic soils, whereas Ca, Zn and Sr can be considered as chemical markers of tomatoes from Scicli, Pachino and Ispica. The results confirm the strong relationship between the quality of agri-food products and their territory of origin, highlighting, on the other hand, the complexity of the system under study (plant-man-environment). Like a complex system, the correlation between food quality and territory of origin can be sometime unpredictable, as it can be affected by exogenic events or by more invasive practices altering the nutritional composition of the fruit, compromising the natural equilibrium between plant and environment. Such unforeseeable events, generating “outliers” samples (well treated by the present multivariate approach), also make it difficult to authenticate products, as evidenced by our results. The XRF technique exhibits high reliability in detecting the chemical elements present in the tomato samples regardless of the instrumental conditions and the type of sample analyzed (pulp with and without skin). The presence of the skin made it possible to obtain a better discrimination of the samples, due to the greater presence in it of some trace elements, such as Ca and Sr, characterizing the tomato fruit composition. According to this finding, including all the parts of fruit in the analysis leads to a better characterization, due to the inclusion of a larger number of in-trace elements.

The elemental signature, thanks to which we were able to uniquely associate the tomato samples to their territory of origin, was mainly highlighted by the analysis of the full data-sets of spectra including the background signal. The spectral analysis carried out as a whole, minimizing the filtering related to the identification of the chemical elements, allowed to better reveal the clustering within a non-homogeneous set of samples, thanks to the inclusion of a more reliable and complete spectrometric information, enriched by the contribution of microelements present in negligible concentration (often removed by background subtraction). The possibility of discriminating fruit and vegetables on the basis of their elemental profiles through rapid and non-destructive analysis techniques, such as the XRF technique, has very important implications in the agri-food sector, which result in large-scale applications of XRF spectrometry aimed at controlling the food quality, preventing food counterfeiting. Moreover, the use of the full data-sets of spectra, including the background, simplify the analysis protocol, making information easily accessible by people not specialized in quantitative XRF analysis, as it allows to bypass the critical procedures related to the background subtraction leading to the extraction of the element yields. Finally, these results corroborate the application of the XRF measurements directly on-the-field, bypassing the sample preparation procedure and the quantitative analysis of the spectra. At present, we have carried out some preliminary measurements on fresh tomato fruit, not dried, which give solid perspectives for a future use of this technique in all the stages of the food chain (production, processing and marketing), supporting the traceability system. In fact, the spectra obtained from freshly picked tomato fruits contained the same characterizing and trace elements identified in the dried “Pulp + Skin” samples. Further analyses are in progress. In the future, we will extend the analysis on cherry tomato samples produced in other agricultural areas of Sicily, as initial step towards the implementation of a database to be used for a fast and non destructive on-the-field fruit identification.

### Supplementary Information

Supplementary Information.

## Data Availability

The data-sets used and/or analysed during the current study available from the corresponding author on reasonable request.

## References

[1] Barchitta M (2017). Integrated approach of nutritional and molecular epidemiology, mineralogical and chemical pollutant characterization: The protocol of a cross-sectional study in women. BMJ Open.

[2] Natesh H, Abbey L, Asiedu SK (2017). An overview of nutritional and antinutritional factors in green leafy vegetables. Hortic. Int. J..

[3] Gallardo H (2016). Possibilities of low-power X-ray fluorescence spectrometry methods for rapid multielemental analysis and imaging of vegetal foodstuffs. J. Food Compos. Anal..

[4] Panebianco S (2022). Feasibility study of tomato fruit characterization by fast XRF analysis for quality assessment and food traceability. Food Chem..

[5] Melquiades FL, Bortoleto GG, Marchiori LFS, Bueno MIMS (2012). Direct determination of sugar cane quality parameters by X-ray spectrometry and multivariate analysis. J. Agric. Food Chem..

[6] Da-Col JA, Bueno MIMS, Melquiades FL (2015). Fast and direct Na and K determination in table, marine, and low-sodium salts by X-ray fluorescence and chemometrics. J. Agric. Food Chem..

[7] Galvan D (2023). Energy-dispersive X-ray fluorescence combined with chemometric tools applied to tomato and sweet pepper classification. Food Control.

[8] Potortì A (2022). Multielement and chemometric analysis for the traceability of the Pachino protected geographical indication (PGI) cherry tomatoes. Food Chem..

[9] Fiamegos Y, Dumitrascu C, Ghidotti M, de la Calle Guntiñas MB (2020). Use of energy-dispersive X-ray fluorescence combined with chemometric modelling to classify honey according to botanical variety and geographical origin. Anal. Bioanal. Chem..

[10] Fiamegos Y, Dumitrascu C, Papoci S, de la Calle MB (2021). Authentication of PDO paprika powder (Pimenton de la Vera) by multivariate analysis of the elemental fingerprint determined by ED-XRF. A feasibility study. Food Control.

[11] Ghidotti M, Fiamegos Y, Dumitrascu C, de la Calle MB (2021). Use of elemental profiles to verify geographical origin and botanical variety of Spanish honeys with a protected denomination of origin. Food Chem..

[12] Lai H (2020). Multi-elemental analysis by energy dispersion X-ray fluorescence spectrometry and its application on the traceability of soybean origin.. At. Spectrosc..

[13] Lia F, Mangion MZ, Farrugia C (2020). Application of elemental analysis via energy dispersive X-ray fluorescence (ED-XRF) for the authentication of Maltese extra virgin olive oil. Agriculture.

[14] Fiamegos Y (2021). Are the elemental fingerprints of organic and conventional food different? ED-XRF as screening technique. J. Food Compos. Anal..

[15] Zdiniakova S, de la Calle MB (2020). Feasibility study about the use of element profles determined by ED-XRF as screening method to authenticate coconut sugar commercially available. Eur. Food Res. Technol..

[16] ISTAT. Crops: areas and production—Fresh vegetables under protective cover (data by provinces). Ist. Naz. di Statistica, Rome, Italy. http://dati.istat.it/Index.aspx?QueryId=37850 &lang=en (2022).

[17] Selvaggi R, Valenti F, Pecorino B, Porto SMC (2021). Assessment of tomato peels suitable for producing biomethane within the context of circular economy: A GIS-based model analysis. Sustainability.

[18] Ministero dell’Agricoltura, della Sovranitá alimentare e Foreste. Disciplinare di produzione dei prodotti DOP, IGP e STG riconosciuti per il settore: Ortofrutticoli e cereali, freschi e trasformati. Disciplinare di produzione della Indicazione Geografica Protetta Pomodoro di Pachino, PQAI IV. https://www.politicheagricole.it/flex/cm/pages/ServeBLOB.php/L/IT/IDPagina/3343#main (2016).

[19] Panebianco S (2022). Epiphytic and endophytic microorganisms associated to different cultivar of tomato fruits in greenhouse environment and characterization of beneficial bacterial strains for the control of post-harvest tomato pathogens. Int. J. Food Microbiol..

[20] Solé V, Papillon E, Cotte M, Walter P, Susini J (2007). A multiplatform code for the analysis of energy-dispersive X-ray fluorescence spectra. Spectrochim. Acta Part B At. Spectrosc..

[21] MathWorks. Matlab (r2020a). The MathWorks, Inc. Natick, Massachusetts, United States. https://www.mathworks.com (2020).

[22] Massart DL, Vandeginste B, Deming S, Michotte Y, Kaufman L (1988). Chemometrics: A Textbook.

[23] Sacco D (2009). Discrimination between Southern Italy and foreign milk samples using spectroscopic and analytical data. Food Chem..

[24] Buccolieri G, Buccolieri A, Donati P, Marabelli M, Castellano A (2015). Portable EDXRF investigation of the patinas on the Riace Bronzes. Nucl. Instrum. Methods Phys. Res. Sect. B Beam Interact. Mater. At..

[25] Lei M, Wang Y, Guo G, Zhang D, Zao X (2021). The bio-avaliability and accumulation of the trace element in rock–soil–fruit system in carbonatite regions of different stratums: Critical soil factors and transfer models. Sci. Total Environ..

[26] Castillo P (2021). Biogeochemistry of plant essential mineral nutrients across rock, soil, water and fruits in vineyards of Central Chile. Catena.

[27] Grainger C (2021). Vineyard site impact on the elemental composition of Pinot noir wines. Food Chem..

[28] LUCAS 2009. TOPSOIL Data - Land Use/Cover Area frame statistical Survey—Joint Research Centre—European Soil Data Centre (ESDAC). https://esdac.jrc.ec.europa.eu/content/lucas-2009-topsoil-data.

[29] Orgiazzi, A., Ballabio, C., Panagos, P. A. & Jones & Fernández-Ugald, O., LUCAS soil, the largest expandable soil dataset for Europe: A review. *Eur. J. Soil Sci.* **69**, 140. 10.1111/ejss.12499 (2017).

[30] Lumivero. XLSTAT statistical and data analysis solution. https://www.xlstat.com/en (2023).

[31] Costantini, E. *et al.**Soil Map of Italy*. Consiglio per ricerca e la sperimentazione in agricoltura. Ministero delle Politiche Agricole, Alimentari e Forestali. https://esdac.jrc.ec.europa.eu/images/Eudasm/IT/2012Carta_Suoli_Italia.jpg (2012).

[32] Shaheen SM, Tsadilas CD, Rinklebe J (2013). A review of the distribution coefficients of trace elements in soils: Influence of sorption system, element characteristics, and soil colloidal properties. Adv. Colloid Interface Sci..

[33] Herrick J, Wander M, Lal R, Kimble JM, Follett RF, Stewart BA (1997). Relationships between soil organic carbon and soil quality in cropped and rangeland soils: The importance of distribution, composition, and soil biological activity. Soil Processes and the Carbon Cycle.

[34] Baldock J, Marschner P, Rengel Z (2007). Composition and cycling of organic carbon in soil. Nutrient Cycling in Terrestrial Ecosystems.

[35] Schneider, A. Gps visualizer. https://www.gpsvisualizer.com (2003–2019).

[36] Shoji S, Nanzyo M, Dahlgren RA (1993). Volcanic Ash Soils: Genesis, Properties and Utilization.

[37] Egli M (2007). Effect of climate and vegetation on soil organic carbon, humus fractions, allophanes, imogolite, kaolinite, and oxyhydroxides in volcanic soils of Etna (Sicily). Soil Sci..

[38] Sharma A, Weindorf D, Wang D, Chakraborty S (2015). Characterizing soils via portable X-ray fluorescence spectrometer: 4. Cation exchange capacity (CEC). Geoderma.

[39] Liu J (2020). Soil organic matter and silt contents determine soil particle surface electrochemical properties across a long-term natural restoration grassland. Catena.

[40] Kabata-Pendias A (2000). Trace Elements in Soils and Plants.

[41] Brusca L (2001). Geochemical mapping of magmatic gas-water-rock interactions in the aquifer of Mount Etna volcano. J. Volcanol. Geotherm. Res..

[42] Nagajyoti P, Lee K, Sreekanth T (2010). Heavy metals, occurrence and toxicity for plants: A review. Environ. Chem. Let..

[43] Falcone E (2021). Impact of acidic volcanic emissions on ash leaching and on the bioavailability and mobility of trace metals in soils of Mt. Etna. Ital. J. Geosci..

