# Supplementary material for: XRF analysis searching for fingerprint elemental profile in south-eastern Sicily tomatoes
Source: Sci Rep. 2023 Aug 23;13:13739. doi: 10.1038/s41598-023-40124-6 (PMC10447457; doi:10.1038/s41598-023-40124-6)
Supplement: Supplementary file 1 — Supplementary Information. [file 41598_2023_40124_MOESM1_ESM.pdf]

SUPPLEMENTARY MATERIAL

XRF analysis searching for fingerprint elemental profile in South-Eastern Sicily tomatoes

Salvina Panebianco[1,2], Maria Grazia Pellegriti[3], Claudio Finocchiaro[4], Agatino Musumarra[1,3,*], Germana Barone[4], Maria Cristina Caggiani[4], Gabriella Cirvilleri[2], Gabriele Lanzafame[4], Alfredo Pulvirenti[5], Agata Scordino[1,6] and Paolo Mazzoleni[4]

[1] Dipartimento di Fisica e Astronomia, Università di Catania, Italy

[2] Dipartimento di Agricoltura, Alimentazione e Ambiente, Università di Catania, Italy

[3] Istituto Nazionale di Fisica Nucleare, Sezione di Catania, Italy

[4] Dipartimento di Scienze Biologiche, Geologiche e Ambientali, Università di Catania, Italy

[5] Dipartimento di Medicina Clinica e Sperimentale, Unità Bioinformatica, Università di Catania, Italy

[6] Istituto Nazionale di Fisica Nucleare, Laboratori Nazionali del Sud, Catania, Italy

[*] Corresponding author

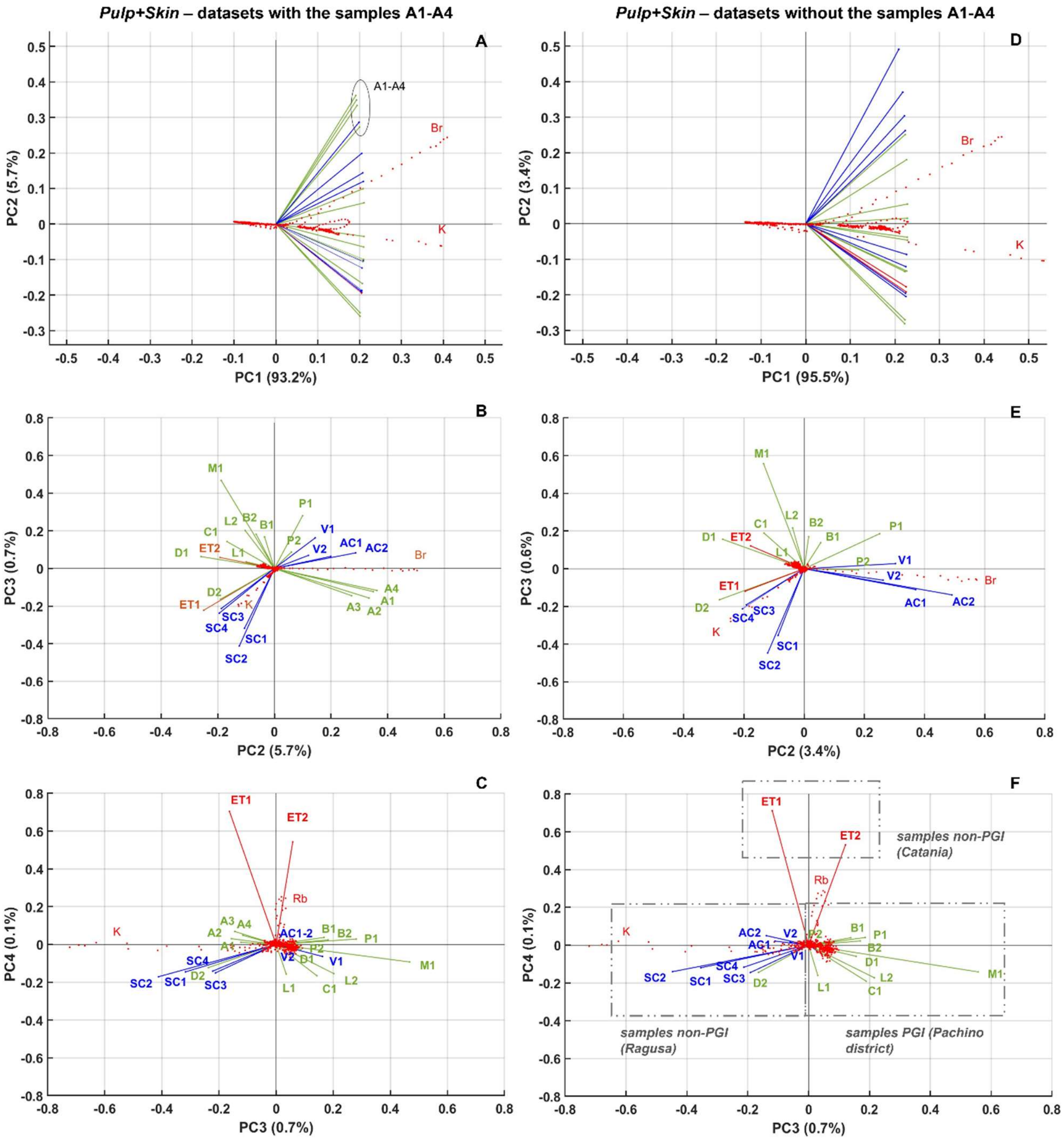

Figure S1. PCA analysis carried out on “Pulp + Skin” samples obtained from tomatoes with PGI and non-PGI certification. The figure shows the comparisons between biplots obtained from datasets consisting of 24 samples, which included the samples A1, A2, A3, A4 from the greenhouse 1 (left side) and from datasets consisting of 20 samples, which excluded the samples A1, A2, A3, A4 from the greenhouse 1 (right side). Datasets were made by tomato XRF spectra that included the background signal. The variables (vectors) symbolizing tomato samples from PGI- and non-PGI sites were distinguished with different colours (green = PGI samples from Pachino district (Ispica and Pachino); blue = non-PGI samples from Ragusa (Acate, Scicli and Vittoria); red = non-PGI samples from Aci S. Antonio). The area of origin and the sample ID were reported in Table 1.

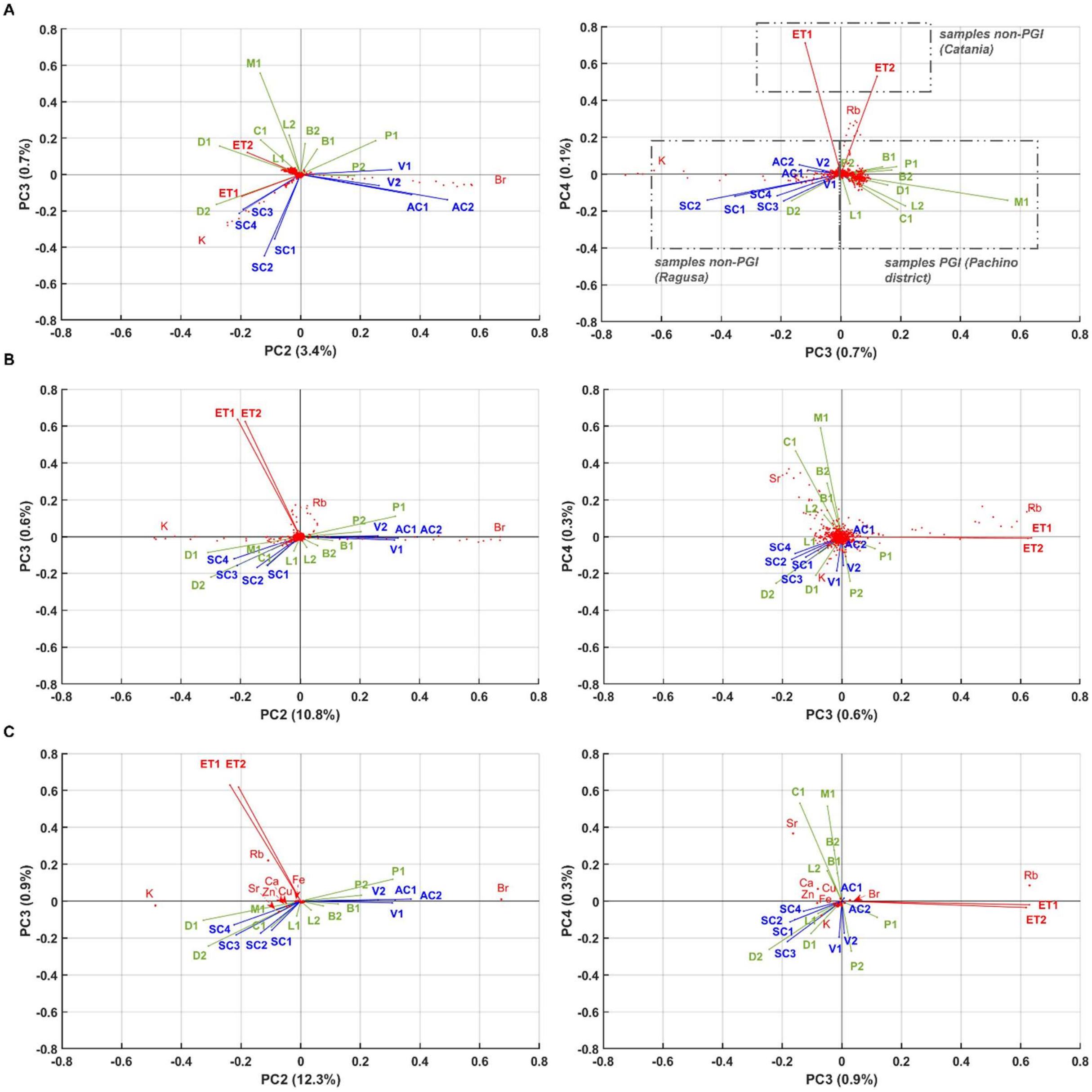

Figure S2. PCA analysis carried out on "Pulp + Skin" samples obtained from tomatoes with PGI and non-PGI certification. Datasets do not include the samples coming from greenhouse 1 (A1- A4). The figure shows the comparisons between the biplot PC2 vs PC3 and PC3 vs PC4 obtained by analyzing "original spectra" with background (A), "net spectra" without background (B) and "element yields" (C). The variables (vectors) symbolizing tomato samples from PGI- and non-PGI sites were distinguished with different colours (green = PGI samples from Pachino district (Ispica and Pachino); blue = non-PGI samples from Ragusa (Acate, Scicli and Vittoria); red = non-PGI samples from Aci S. Antonio). The area of origin and the sample ID were reported in Table 1.

Table S1. Correlation matrix for the variables used in the Discriminant Analysis (DA).

Variables	Cl	K	Ca	Fe	Cu	Zn	Br	Rb	Sr
Cl	**1**	0.328	0.353	0.154	-0.122	-0.214	-0.230	0.318	0.180
K	0.328	**1**	0.396	0.753	0.487	0.692	-0.372	0.033	0.194
Ca	0.353	0.396	**1**	0.425	0.243	0.326	-0.564	-0.100	0.554
Fe	0.154	0.753	0.425	**1**	0.444	0.497	-0.149	0.189	-0.029
Cu	-0.122	0.487	0.243	0.444	**1**	0.705	-0.340	0.172	0.383
Zn	-0.214	0.692	0.326	0.497	0.705	**1**	-0.274	-0.264	0.367
Br	-0.230	-0.372	-0.564	-0.149	-0.340	-0.274	**1**	-0.312	-0.423
Rb	0.318	0.033	-0.100	0.189	0.172	-0.264	-0.312	**1**	-0.101
Sr	0.180	0.194	0.554	-0.029	0.383	0.367	-0.423	-0.101	**1**

Table S2. Classification functions intercepts obtained by using the Discriminant Analysis (DA).

	Acate	Aci St. Antonio	Ispica	Pachino	Scicli	Vittoria
Intercept	-225.488	-1148.503	-180.643	-67.501	-99.633	-177.500
Cl	-231.110	573.816	-170.774	-31.025	98.769	-174.789
K	12.024	-23.267	10.847	3.488	-1.314	9.819
Ca	-19.996	66.770	-13.554	0.656	18.370	-15.502
Fe	-433.734	919.064	-321.326	-100.800	152.292	-355.005
Cu	272.140	-480.060	161.206	91.884	-96.119	254.063
Zn	98.789	-254.415	107.338	16.335	-26.878	67.791
Br	8.207	-17.671	5.100	1.792	-3.164	6.706
Rb	-72.322	187.041	-46.158	-10.222	32.005	-58.190
Sr	16.420	-45.828	10.391	1.583	-8.113	11.495

Table S3. Prior and posterior classification into groups, membership probabilities (Pr) and squared distances (D^2) obtained, for each observation, by using the Discriminant Analysis (DA).

Observations [a]	Prior	Posterior	Pr (Acate)	Pr (Aci St. Ant.)	Pr (Ispica)	Pr (Pachino)	Pr (Scicli)	Pr (Vittoria)	D² (Acate)	D² (Aci St. Ant.)	D² (Ispica)	D² (Pachino)	D² (Scicli)	D² (Vittoria)
B1	Pachino	Pachino	0.00	0.00	0.00	1.00	0.00	0.00	317.45	2332.38	209.25	10.51	149.44	199.05
B2	Pachino	Pachino	0.00	0.00	0.00	1.00	0.00	0.00	255.86	2505.83	165.02	9.43	183.46	157.67
C1	Pachino	Pachino	0.00	0.00	0.00	1.00	0.00	0.00	295.95	2466.44	174.39	14.53	173.02	186.42
M1	Pachino	Pachino	0.00	0.00	0.00	1.00	0.00	0.00	229.21	2650.74	126.45	13.25	227.80	137.02
P1	Pachino	Pachino	0.00	0.00	0.00	1.00	0.00	0.00	281.55	2458.12	179.03	15.00	200.58	172.42
P2	Pachino	Pachino	0.00	0.00	0.00	1.00	0.00	0.00	266.23	2530.52	144.05	14.75	170.78	162.44
D1	Ispica	Ispica	0.00	0.00	1.00	0.00	0.00	0.00	148.82	3479.16	12.93	143.16	476.79	115.06
D2	Ispica	Ispica	0.00	0.00	1.00	0.00	0.00	0.00	149.91	3686.32	12.52	189.20	512.15	135.09
L1	Ispica	Ispica	0.00	0.00	1.00	0.00	0.00	0.00	106.00	3634.51	8.96	156.29	492.40	101.09
L2	Ispica	Ispica	0.00	0.00	1.00	0.00	0.00	0.00	134.37	3630.33	11.06	170.62	502.20	135.40
ET1	Aci St. Antonio	Aci St. Antonio	0.00	1.00	0.00	0.00	0.00	0.00	4364.99	4.85	3651.84	2521.43	1597.56	3871.04
ET2	Aci St. Antonio	Aci St. Antonio	0.00	1.00	0.00	0.00	0.00	0.00	4270.40	4.85	3550.28	2443.79	1533.06	3781.60
AC1	Acate	Acate	1.00	0.00	0.00	0.00	0.00	0.00	8.62	4159.73	85.51	227.40	721.22	30.84
AC2	Acate	Acate	1.00	0.00	0.00	0.00	0.00	0.00	8.62	4483.20	178.54	312.76	889.16	37.68
V1	Vittoria	Vittoria	0.00	0.00	0.00	0.00	0.00	1.00	28.12	3936.01	123.91	183.01	666.23	6.97
V2	Vittoria	Vittoria	0.00	0.00	0.00	0.00	0.00	1.00	37.12	3720.88	110.62	143.46	590.72	6.97
SC1	Scicli	Scicli	0.00	0.00	0.00	0.00	1.00	0.00	823.54	1542.02	520.41	182.38	9.09	639.83
SC2	Scicli	Scicli	0.00	0.00	0.00	0.00	1.00	0.00	864.09	1514.23	545.53	209.18	8.20	682.52
SC3	Scicli	Scicli	0.00	0.00	0.00	0.00	1.00	0.00	731.58	1659.25	432.49	149.22	8.55	565.14
SC4	Scicli	Scicli	0.00	0.00	0.00	0.00	1.00	0.00	800.94	1560.20	473.49	178.16	8.02	632.37

[a] The observations represent element yields normalized to Ar yield, detected in the "Pulp + Skin" samples under study. The samples were obtained from tomatoes with PGI and non-PGI certification harvested in Pachino (P1, P2, B1, B2, C1, M1), Ispica (D1, D2, L1 and L2), Aci St. Antonio (ET1, ET2), Acate (AC1, AC2), Vittoria (V1, V2) and Scicli (SC1, SC2, SC3 and SC4) (see Table 1).
